# Rooted triple consensus and anomalous gene trees

**DOI:** 10.1186/1471-2148-8-118

**Published:** 2008-04-25

**Authors:** Gregory B Ewing, Ingo Ebersberger, Heiko A Schmidt, Arndt von Haeseler

**Affiliations:** 1 Center for Integrative Bioinformatics Vienna, Max F. Perutz Laboratories, Dr. Bohr Gasse 9, A-1030 Vienna, Austria

## Abstract

**Background:**

Anomalous gene trees (AGTs) are gene trees with a topology different from a species tree that are more probable to observe than congruent gene trees. In this paper we propose a rooted triple approach to finding the correct species tree in the presence of AGTs.

**Results:**

Based on simulated data we show that our method outperforms the *extended majority rule consensus *strategy, while still resolving the species tree. Applying both methods to a metazoan data set of 216 genes, we tested whether AGTs substantially interfere with the reconstruction of the metazoan phylogeny.

**Conclusion:**

Evidence of AGTs was not found in this data set, suggesting that erroneously reconstructed gene trees are the most significant challenge in the reconstruction of phylogenetic relationships among species with current data. The new method does however rule out the erroneous reconstruction of deep or poorly resolved splits in the presence of lineage sorting.

## Background

How a species relates with one another is of fundamental importance in evolutionary biology. The reconstruction of these relationships among species is now a problem frequently approached with biological sequence data [1-4]. Unfortunately this has not necessarily improved the clarity of species relationships and species trees remain a topic of debate [5]. In many respects the use of molecular data has meant that significantly more complicated models of evolution must be considered. Consequently, reconstructing species trees from gene trees has to cope with two classes of difficulties. One class relates to the problem of accurate tree reconstruction from biological sequences. This is due to both the well known Felsenstein zone type problems [6,7] caused by long branch attraction, and the limited amount of phylogenetic signal in finite sequences [8-10]. The second class is comprised by the effects of lineage sorting resulting in a genealogy different from that of the species [11,12]. Both kinds of problems are known to gain severity when the length of internal branches in a species tree becomes small.

The most prominent example where a short internal branch interferes with the conclusive reconstruction of genetic relationships between species are humans, chimpanzees, and gorillas [13-15]. However, even in the presence of lineage sorting, for 3 species the most probable gene tree represents the species tree [16]. Thus, the problem of the evolutionary ancestry of humans, chimpanzees and gorillas was eventually approached by sampling a large number of significantly resolved gene trees from different loci and accepting the most frequently observed topology tree as the species tree [13,17,18]. It is now common practice to reconstruct gene trees from several genomic loci, e.g., with Maximum Likelihood methods [19]. The resulting trees are then combined into a bona fide species tree with one of several consensus methods [20,21], e.g. the 50% majority consensus (M_50%_), the Majority Rule Extended (MRe) [22], or the Relative Majority Consensus [23]. Alternatively, all sequences can be concatenated first, and then a single tree is reconstructed that is hoped to reflect the species' evolutionary relationships. The latter approach resembles a consensus method where a weighted phylogenetic signal is used to estimate the consensus tree [21,24,25]. Recently, however, it was shown that with 4 taxa the most probable gene tree does not necessarily reflect the species tree, if the species tree is unbalanced [16]. Such *anomalous gene trees *(AGTs) [16] can occur since not all tree topologies are equi-probable under a coalescent model [16,26,27]. This effect becomes more severe when trees with five or more taxa are considered. [28,29]. AGTs were proven to exist for *n*-maximal probable species trees with 5 or more taxa, where a species topology with *n *taxa is defined to be *n-maximal probable *if its probability under the Yule model [30-32] is maximal. Consequently, all species trees with 5 or more taxa can produce AGTs. Therefore, existing majority rule consensus methods can be statistically inconsistent with a coalescent model of evolution. Positively misleading results can also be obtained when sequences are concatenated to arrive at a consensus tree [33].

A recent example where AGTs potentially interfere with the reconstruction of the correct species tree is the reconstruction of the metazoan phylogeny. Evidence exists for radiation events during early metazoan evolution [34]. Accordingly, Felsenstein zone type problems connected to the proposed radiation events were said to hinder an accurate gene tree, and thus species tree reconstruction [34]. However, the potential effect of AGTs, which are also likely to arise in phylogenies with long external and short internal branches was not taken into account.

**Figure 1:**
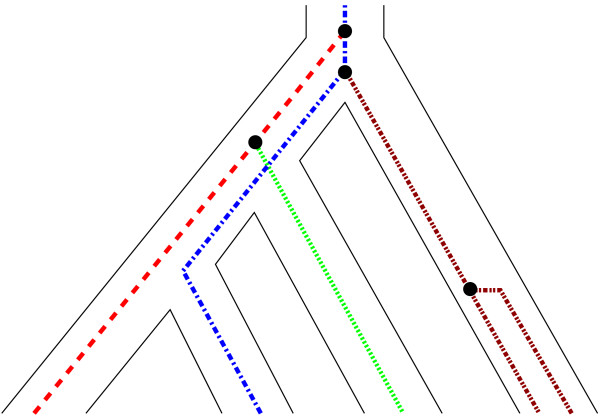
**Coalescent speciation model**. An illustration of the coalescent speciation model. Note that a coalescent between distinct species must occur further into the past than the speciation event for incongruent gene trees to exist.

The correlation between the genealogy of the compared sequences and that of the corresponding species is usually modelled by a coalescent process [12,28,29]. The underlying Kingman coalescent model [35,36] is used with two basic assumptions. At a speciation event (internal node on the species tree; Figure 1) travelling past to present, the population splits into two isolated populations. Furthermore, all species (past and present) have a constant population size. Now sampling a number of alleles from different individuals of a single species and tracing the genetic lineages backward in time, we have a traditional Kingman coalescent process with exponential waiting time between gene coalescent events within a species [28,35,36]. The rate with which two genetic lineages in a population coalesce is proportional to 1/*θ *where *θ *is a measure of the effective population size. If we continue to go backwards in time past the next speciation event, genetic lineages from the related species are added to the process (c.f. Figure 1). By that, genetic lineages from species that share this ancestral population can coalesce, giving rise to the gene tree. As *θ *→ 0 a gene tree will have the same topology as the species tree with probability close to 1 since all genetic lineages in a population will coalesce before genetic lineages from a different species are added. However, when *θ *→ ∞ all coalescent events will occur above the root of the species tree. Therefore, gene trees are not necessarily correlated to the species tree. In simple terms, a large *θ *gives rise to a larger proportion of incongruent gene trees, an effect that has been described as lineage sorting [11,12].

Currently, two general alternatives exist to assess the effect of AGTs on phylogeny reconstruction. One requires the addition of more sequences from the same locus for each species or clade of interest. This approach relies on the fact that these particular sequences coalesce after the speciation event of interest [37]. It will, therefore, not aid AGTs caused by divergences deep in the phylogeny since all the lineages from a particular taxon coalesce with high probability before the divergence of the taxa under study. The second general approach makes full use of the coalescent model with the speciation process. A likelihood function can be derived over a set of gene trees and a species tree [38]. A Maximum Likelihood or Bayesian method can then be used to estimate all the trees and other relevant parameters. This approach seems promising for smaller data sets [39-41]. However, it carries a number of drawbacks. For example, removing the molecular clock assumption is not trivial. Furthermore, the population parameter will likely not be constant in time or across species. The latter problem was addressed in [42] with a Bayesian approach by modelling correlation between gene trees with a prior rather than with a coalescent model. The above methods provide a first approach to dealing with the AGT problem. However, they share the limitation of becoming computationally prohibitive with the large numbers of taxa and loci that are considered.

Here we present a new and fast consensus method to reconstruct a species tree from a set of gene trees that is insensitive to the AGT problem. Using simulations, we show that our method outperforms traditional consensus methods in reconstructing the correct species tree in the presence of AGTs. Eventually, we apply our program to assess the potential influence of AGTs on the reconstruction of the metazoan phylogeny.

## Results and Discussion

The approach we take to reconstruct a correct species tree in the presence of AGTs is based on the observation that rooted three taxa trees do not exhibit AGTs [28,29]. For a given set of sequence alignments, we first estimate the individual gene trees using traditional phylogenetic methods. We then extract the $\left(\begin{array}{c} n\\ 3 \end{array}\right)$ rooted three taxa trees from each gene tree and take the most frequently occurring as the species triplet tree. The set of rooted triples is then combined to produce a species tree using the QUARTET-PUZZLE heuristic [43]. The details of the method are presented in Section METHODS AND MATERIALS. The main advantages of the method are that there are no requirements to estimate any coalescent parameters, it is fast, eliminates AGTs regardless of the coalescent history, and scales well to larger problems. We refer to this method as the Triple Construction Method (TCM).

The intention of our simulations is to demonstrate the performance we might expect from real data. So rather than using a tree that is artificially in the AGT zone and comparing results, we use a Yule tree prior [30,32] on species trees and compare performance of the respective methods over this prior. Other priors are possible, however we believe that the Yule process models speciation with sufficient accuracy for this study [31,32]. We do not attempt to measure the tree "distance" from the true tree, and report only full correct reconstructions. Finally we do not report M_50% _consensus results because for the parameters of interest the M_50% _will not resolve species trees. Also if the M_50% _method does resolve species tree both TCM and MRe will produce identical species trees. Therfore we compare TCM with MRe.

### Simulation Results

We first applied our method to simulated data. For all simulations species trees were generated from a Yule process with a birth rate of 5. Gene trees were then simulated from the species trees using a coalescent model [12,28,29]. These gene trees were subsequently used to reconstruct the species tree with two methods, our TCM and the Majority Rule extended (MRe) [22]. To check for correctness of our method, we tested it first on the smallest size species tree where AGTs can exist (4 taxa; data not shown). We then extended the simulation to 20 taxa, five different *θ*-values and 8 data sets ranging from 10 to 10,000 loci in size. The results are shown in Table 1. For almost all parameters considered TCM performs at least equal but in most cases better than MRe. With the smallest *θ*-value the advantage of TCM over MRe is the least prominent. This is a reflection of the low number of anomalous gene trees expected with low values of *θ*. The difference in performance of the two methods, however, becomes more obvious with larger *θ *values. The number of correctly reconstructed species trees with TCM increases with increasing numbers of genes, while the performance of MRe does not benefit to the same degree and sees little improvement with very large numbers of loci. The high number of genes were chosen to confirm this asymptotic performance of TCM. This difference in accuracy between the two methods is the behaviour we would expect in the presence of AGTs as there is a nonzero probability of the Yule process generating a species tree that will give rise to AGTs.

**Table 1:** Simulation Results. Simulation results for the reconstruction of a 20 taxa tree over a range of *θ *values and numbers of genes. For all parameter combinations 1000 replicates were performed. Numbers reflect the percentage of correctly inferred species trees. Dashes indicate simulations that were not run due to their very low levels of congruent gene trees. TCM is the Triple Construction Method and MRe is Majority Rule Extended.

		Number of Genes							
*θ*	Method	10	50	100	200	500	1000	2000	10000
0.01	TCM	79	85	87	90	92	96	98	98
0.01	MRe	80	84	85	89	90	95	98	98
0.05	TCM	36	52	63	71	77	86	93	96
0.05	MRe	36	52	63	71	76	80	86	87
0.1	TCM	16	27	39	45	60	69	82	91
0.1	MRe	14	25	36	40	49	54	61	66
0.2	TCM	-	-	-	33	48	61	67	82
0.2	MRe	-	-	-	21	29	31	36	38
0.5	TCM	-	-	-	11	18	26	41	63
0.5	MRe	-	-	-	3	4	6	6	8

The type of species tree that can give rise to AGTs is also the type of tree that potentially causes problems with correct gene tree reconstruction. In particular effects that bias topologies such as long branch attraction may also produce a bias on the derived species tree with different consensus methods. To assess the effect of gene tree reconstruction errors, we used the simulated gene trees to generate short (200 nucleotides) simulated alignments. From theses alignments we inferred the maximum likelihood gene trees, which were subsequently used to reconstruct the species' phylogeny. The results for both reconstruction methods TCM and MRe are presented in Table 2. With small sized data sets (10 – 20 loci), incorrectly reconstructed gene trees interfere substantially with correct species tree reconstruction. However, when the number of loci increases, the performance recovers quickly and is only slightly reduced compared to the scenario with no gene tree reconstruction errors. Notably, the performance of both methods degrades approximately to the same extent. We therefore conclude that phylogenetic reconstruction errors in gene trees add a form of unbiased noise to the species tree reconstruction problem.

**Table 2:** ML Simulation Results. Simulation results with maximum likelihood tree reconstruction. Each gene tree was reconstructed with phyML with 200 sites with a GTR nucleotide substitution model. In all cases there were 20 taxa per tree and 1000 replicates.

		Number of Genes						
*θ*	Method	10	50	100	200	500	1000	2000
0.05	TCM	8	36	53	66	75	80	86
0.05	MRe	9	35	48	64	74	81	83
0.1	TCM	6	15	36	47	63	74	81
0.1	MRe	6	14	31	38	51	65	69

Another important parameter to consider during tree reconstruction is the number of taxa on the tree. Under a Yule speciation process, larger species trees have an increased chance of containing sub trees with the required short branches necessary for AGTs to occur. The results from simulations with increasing species tree size is shown in Table 3. The number of genes is 200, 500 and 1000 with *θ *= 0.1. With small numbers of taxa both methods have similar performance. However, as the number of taxa increases the performance of TCM clearly outperforms MRe. The accuracy drops for both methods quite quickly with larger numbers of taxa. Again we note the increase in performance with larger numbers of loci. This indicates that even with quite low *θ *AGTs could still be a problem with large trees.

**Table 3:** Variable taxa results. Simulation results for varying number of taxa with *θ *= 0.1 and 200, 500 and 1000 Genes.

Number Genes	Method	Number of Taxa							
		5	10	15	20	25	30	35	40
200	TCM	90	70	55	45	41	31	27	17
200	MRe	89	64	51	40	32	29	20	15
500	TCM	92	76	73	60	56	48	41	34
500	MRe	91	72	62	49	45	39	30	22
1000	TCM	95	82	81	69	65	63	56	50
1000	MRe	95	76	66	54	53	42	40	31

### Metazoa Data

We then assessed whether AGTs are an issue in the reconstruction of phylogenies from biological sequence data. The reconstruction of the animal phylogeny was chosen as an example. We compiled a set of 216 orthologous proteins from 20 metazoan species and yeast. Protein alignments were produced with T-coffee [44] using the default parameter settings. All gene trees were reconstructed with phyML [45]. We used the I+Γ JTT model of protein evolution with 4 rate categories, where both the proportion of invariant sights and the Γ shape parameter were estimated from the data.

**Figure 2:**
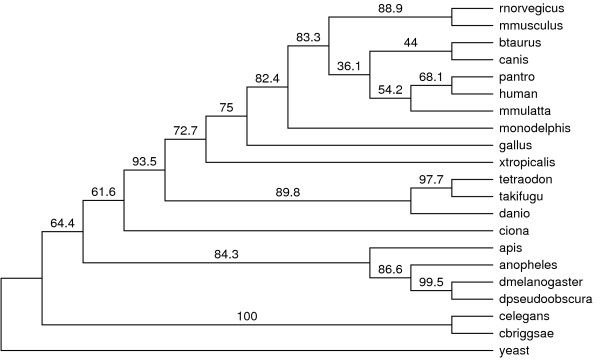
**Metazoa Tree**. The tree from the metazoa data showing the support in number of genes for the given topology. There are a total of 216 genes and we note that only two branches have less than 50% support.

The species phylogeny derived from the 216 genes with the yeast outgroup removed and obtained with the TCM method is shown in Figure 2. The tree has good support and the clade with the least support (*Canis familiaris *and *Bos taurus*) is still supported by more than 36% of the gene trees. Comparing the TCM species tree topology with that obtained with other consensus methods (M_50% _and MRe) shows that all methods arrive at the same conclusion.

It is noted that the nematodes are placed basal to the arthropods with greater than 50% support. This would lend support to the hypothesis that animals with a central body cavity form the monophyletic group of *Coelomata *[46,47]. However, it contrasts the wide spread belief that places nematodes and arthropods in the monophyletic clade of *Ecdysozoa *[48,49]. Proponents of the Ecdysozoa hypothesis claim that the basal position of *C. elegans *is an artifact of phylogenetic reconstruction errors due to long branches and poor taxon sampling. In fact, when inspecting our gene trees, many had very long branches (greater than one expected substitution per site). Thus, saturation of the phylogenetic signal is a problem and long branch attraction cannot be ruled out. However, resolution of this problem is outside the scope of this paper.

## Conclusion

We have implemented a rooted triple consensus method that is not prone to AGTs and have demonstrated that it performs equal or better than the traditional Majority Rule Consensus method at reconstructing species trees across a range of scales.

It should be noted that with small numbers of genes, error in species tree reconstruction is dominated by stochastic effects for both large and small *θ*.

The effects of maximum likelihood phylogenetic tree reconstruction errors in the gene trees was investigated. It was found that reconstruction accuracy of both methods considered (TCM and MRe) were degraded similarly. We conclude that reconstruction errors do not bias the species tree reconstruction any more in TCM than other consensus methods.

The support an AGT topology can have must be below 1/3 or about 33%. This follows from the fact that if there are just 3 lineages surviving past some speciation event, all three of the possible topologies are equiprobable and hence no AGT. While if we consider 4 lineages surviving into a common species, the most likely topology has probability 1/9. Five or more gene lineages surviving until a recent common ancestor have maximal likelihood gene trees with even lower probabilities [16,26,27]. Once the different cases have been taken into account over a species tree, the total contribution an AGT can have is less than 1/3. This has also been verified with simulations.

A metazoan data set was compiled in order to compare the method with real data. The results indicated that AGTs do not play a role in reconstruction problems of the sizes considered. There was no difference between the construction methods considered (M_50%_, MRe and TCM). In this case we can rule out AGTs when we consider the maximum gene tree support that an AGT can have is less than 33%.

Not presented are a large number of data sets investigated that do not show an AGT signature. In particular high support of typically more than 50% is common in eukaryotes with large numbers of genes [5]. Other examples lack the number of loci to consider AGTs or have species trees of only 3 taxa [41,50]. Furthermore the AGT signature is a very poorly supported clade (¿33% or less) and at this point our models of evolution present large enough uncertainties that we would not back such a clade with confidence. For example [18] although it had both sufficient numbers of taxa and loci, we only considered the data which could produce statistically distinguishable trees.

Referring to a recent study on the metazoan species tree [34] we note that much of the tree has high support and furthermore they demonstrate the strong dependence of phylogenetic reconstruction error on this tree. In fact a majority of studies use a very conservative threshold when determining species trees and prefer to leave the tree unresolved.

Another reason for the current lack of AGT signatures in the data may be due to the fact that a species tree wide effective population parameter (*θ*) is small. Consider a speciation event caused by geographical isolation (eg at the last glacial maximum). At the time of isolation the population size is much smaller than the original population of the founding species. The net result is that the effective population can be very small and the likelihood of AGTs also correspondingly small. However much larger species trees and larger numbers of sampled genes will lead to data sets in the future that AGTs will need to be considered.

In the case that AGTs are present M_50% _would never fully resolve the species tree and MRe would reconstruct an incorrect species tree. Our method resolves these issues without a large computational burden for larger data sets with large numbers of species and loci. If rooted topologies or a good outgroup is available, and M_50% _does not resolve the species tree adequately, then TCM should be used in preference to MRe. As bigger data sets become available and as biologists desire to resolve larger species trees that all diverged at similar times, we believe a TCM approach to species tree reconstruction will become an important tool.

## Methods

First a species tree is generated from a Yule prior or a constant rate birth process with no extinction. Then a number of gene trees are generated from this species tree. For some simulations DNA alignments are then generated from the gene trees. The maximum likelihood method phyML [45] was used to estimate the phylogenies from the sequence data. In our case we do not reconstruct the phylogeny with a clock although the underlying coalescent model would produce only clocklike trees. In order to locate the root we add a outgroup for all sequence data. This outgroup is used to form rooted three taxa trees, that is, quartets where one taxon in the quartet is always the outgroup. For most of the simulations we do not include gene tree reconstruction for performance reasons and the generated gene trees are used directly by the estimating methods. This represents the ideal performance of these methods.

Once the gene trees were generated or found, each $\left(\begin{array}{c} n\\ 3 \end{array}\right)$ rooted triplet tree was considered across all gene trees for the dataset. That is, for each set of three taxa the majority rule was used to decide which of the three topologies to select. After this step there are $\left(\begin{array}{c} n\\ 3 \end{array}\right)$ rooted three taxa trees that hopefully contain no conflicts if there are sufficient gene trees. In practise however, with real data there will be some conflict. We combine these rooted three taxa trees using the quartet puzzling heuristic [43] the details of which are presented below. The result was compared to the true species tree, and scored correct if and only if it has the same topology. That is, we only consider if the species tree is recovered correctly or not. The MRe consensus method was implemented and compared to our method with several options.

An implementation of root triple consensus program is publicly available at http://www.cibiv.at/software/triplec/ and also [see Additional file 1].

### Tree Puzzle Heuristic

Quartet Puzzling [43] is a simple but effective heuristic for combining potentially conflicting quartets for tree inference. Mapping a quartet method to rooted three taxa trees is straightforward once the root in the reconstructed topology is treated as a special label or taxon with a "virtual" branch from the true root to this "root" taxon. In this way when new taxa are added they can be connected above the current root. This is equivalent to simply using the original quartet puzzling algorithm with an extra taxon (the outgroup) to denote the root on both the quartets used and the reconstructed tree, and we only use the $\left(\begin{array}{c} n\\ 3 \end{array}\right)$ quartets that contain this root label taxon. We now describe the algorithm in detail.

Consider the set of *n *taxa with labels L ∈ {1, 2,...*n*} and the set of $\left(\begin{array}{c} n\\ 3 \end{array}\right)$ triples denoted T ∈ {{*a*, *b*, *c*} : 1 ≤ *a *<*b *<*c *≤ *n*}. Each triple {*a*, *b*, *c*} can form any one of three *rooted *three taxa trees, namely (*a*, *b*|*c*), (*a*, *c*|*b*) and (*b*, *c*|*a*). Here we use the notation that the rooted triplet tree (*a*, *b*|*c*) is equivalent to the Newick formatted tree ((*a*, *b*), *c*) placing the root between *c *and the (*a*, *b*) clade. We consider the rooted triplet tree occurring across all gene tree most frequently as the correct one for any 3 taxa.

We start with the first rooted triplet tree. We now add a single taxon at a time until we have added all the taxa. We add taxon *x *to a tree with the first *x *- 1 taxa already present as follows. For every triple (*a*, *b*, *x*), 1 ≤ *a *<*b *≤ *x *- 1 we inspect the rooted triplet tree. If the rooted triplet tree is (*a*, *b*|*x*), for example, then the taxon cannot be added on any edge between *a *and *b *without conflicting with this rooted triplet tree. Therefore we add a penalty of one on all edges on the path between *a *and *b*. Similarly if the rooted triplet tree was (*a, x*|*b*) we would add a penalty of 1 on all edges between *b *and the root, recalling that there is a "virtual" edge above the root. Once all $\left(\begin{array}{c} x-1\\ 2 \end{array}\right)$ triples have been inspected. The new taxon *x *is added to the edge with the lowest penalty. In the case of a tie we choose which edge to add to randomly. First we note that if the set of rooted triplet trees do not conflict then we will always reconstruct the correct tree. However we also note that if the rooted triplet trees do conflict then the reconstructed tree will depend on the order the taxa are added. Because of this we generate a set of intermediate trees with randomised taxa order, and then take a consensus of this set of trees. The consensus method used is discussed below. The default is to generate 5000 intermediate trees from a set of rooted three taxa trees.

### Consensus Methods

Avoiding unnecessary notation, we can represent any vertex in a rooted topology as a set of all the taxa that are below that vertex. The root therefore is the full set of taxa labels while vertices elsewhere are subsets of their parent vertex. All the tree majority consensus methods start the same way. All the subsets induced by vertices in the rooted trees of interest are ranked by frequency of occurrence. The methods vary in the way these ranked subsets are stitched back together to form a tree.

The method we used with the quartet puzzling is the Relative Majority consensus method [23]. We simply add subsets starting from the most highly supported subset until the first subset conflicts with previously added subsets. A conflicting subset is a subset that cannot be placed anywhere in the tree where it is constrained to be a subset of a parent vertex and all descendant vertices are subsets of itself. Thus, we have a tree that, although may not be fully resolved, only contains vertices which are supported more than any other vertex not in the tree.

In contrast, the MRe method continues to add non-conflicting subsets until the tree is resolved or there are no subsets left. Thus, the consensus tree may contain vertices that are supported less than some contradicting vertex not present in the tree.

## Authors' contributions

AvH conceived the study and contributed to the manuscript. GBE implemented the code, carried out the simulations and prepared the manuscript. HAS contributed to code development and manuscript preparation. IE prepared and interpreted the metazoa data and contributed to the manuscript.

## Supplementary Material

Additional File 1 Triplec. The java implementation of rooted triple consensus. See http://www.cibiv.at/software/triplec/ for further details and updates. Click here for file
